# Invisibility Cloaking Scheme by Evanescent Fields Distortion on Composite Plasmonic Waveguides with Si Nano-Spacer

**DOI:** 10.1038/s41598-017-10578-6

**Published:** 2017-09-21

**Authors:** Yakov Galutin, Eran Falek, Alina Karabchevsky

**Affiliations:** 1 Electrooptical Engineering Unit, Ben-Gurion University of the Negev, Beer-Sheva, 8410501 Israel; 2 Ilse Katz Institute for Nanoscale Science & Technology, Ben-Gurion University of the Negev, Beer-Sheva, 8410501 Israel; 3 Department of Electrical and Computer Engineering, Ben-Gurion University of the Negev, Beer-Sheva, 8410501 Israel

## Abstract

A new, composite plasmonic waveguide based electromagnetic cloaking scheme is proposed with Si nano-spacer. Here we show, that the scattering fields of an object located on the cloak do not interact with the evanescent field, resulting in object’s invisibility. Finite difference time domain (FDTD) numerical calculations were performed to extract the modal distributions and surface intensities on a composite plasmonic waveguide with a metasurface overlayer. Spatially varying effective permittivity was analytically calculated using transformation optics. Cloaking was demonstrated for a cylindrical object with diameter of 70% from the waveguide width on a high index ridge waveguide structure with silicon nitride guiding layer on silica substrate. Our results open the door to new integrated photonic devices, harnessing from evanescent fields distortion on composite plasmonic waveguides and dielectric nano-spacers for the variety of applications from on-chip optical devices to all-optical processing.

## Introduction

The concept of an invisibility cloak with metamaterials has been a topic of interest over the last few centuries. Metamaterials are a man-made engineered materials composites with exotic electromagnetic properties achieved through subwavelength structuring^1–3^. However, obtaining effective material parameters for the metamaterials is not trivial due to their structural inhomogeneity and strong spatial dispersion^4,5^. The recent developments in metamaterial science^6–9^ and nanotechnology^10,11^ have enabled the possibility of cloaking an object to become a technological reality. One approach to achieve an invisibility cloak is transformation optics^12,13^. In this approach, based on the invariance of Maxwell’s equations to coordinate transformations, we can deform the wave paths in a specific manner. Instead of physically transforming the medium, the values of the chosen material parameters are modified, i.e., the relative permittivity and permeability tensors are changed^14^. This leads to a spatially varying medium parameters which, in essence, transform the coordinates of the electromagnetic fields, resulting in a concealed object.

Silicon photonics integrated circuits are considered to enable future computing systems with optical input-outputs co-packaged with CMOS chips to circumvent the limitations of electrical interfaces. Silicon based photonic integrated circuits are widely used in applications such as optical modulators^15^, optical interconnects^16^, biosensors^17,18^, and more. A metamaterial overlayer, or simply a metasurface, on an integrated photonic structure, allows for nurturing the device with novel functionalities. The introduction of metasurfaces simplifies the design of metamaterial structures due to its characteristic thickness, which typically much smaller than the wavelength. This essentially converts the design process to two dimensional. One of the most appealing applications of metasurfaces is achieving invisibility cloaks by tailoring evanescent fields. This can be allowed in a controllable manner using an integrated photonics platform.

Composite plasmonic waveguides^19^ incorporating dielectric and metallic films such as in ref.^19^, offer great potential for ultra-compact integrated photonic devices due to substantial increase of the propagation distance of the plasmon waves. Surface plasmons^20,21^ (SP) are a type of a surface waves extending along a metal-dielectric interface considering the momentum matching conditions. The SP benefits from spatial confinement and high local field intensity. One of the major limitations in the implementation of plasmonic circuits is their short propagation length. Composite plasmonic waveguides^19^ provide large confinement of light in sub-wavelength scale. They also allow for the control of the surface plasmons excited in the metal overlayer while substantially reducing inevitable ohmic losses of conventional plasmonic materials.

In this paper we present an analysis of the modal distribution and surface intensity in a channel photonic waveguides with a metasurface overlayer. The spatial distribution of the metasurface permittivity was analytically calculated based on the transformation optics principles. The spatial distribution was then imported into a commercial Maxwell solver (Lumerical) using finite-difference time-domain method (FDTD).

## Theory and Design

### Composite Plasmonic Waveguide Structure

Our theoretical analysis is based on the semi-analytical model of orthonormalization of complex eigenmodes at an abrupt step, developed for the investigation of the transmission and surface intensity in composite plasmonic waveguide structures^19^. Briefly, the monochromatic plane wave with *λ*_0_ illuminates the waveguide endfacet and excites the fundamental guided mode *E*_*i*0_. At some distance from the waveguide input, there is an abrupt step introduced by the slab plasmonic film made of gold with length of *L* along the propagation direction *z*. This region is called a composite plasmonic waveguide structure since it includes the dielectric waveguide with plasmonic overlayer which excites three orthogonal hybrid plasmonic modes *E*_*γ*1_. The transmittance and surface intensity is obtained by thexpans mode matching at the abrupt steps and the explicit formulation of the eion coefficients which expand modes at abrupt steps.

**Figure 1:**
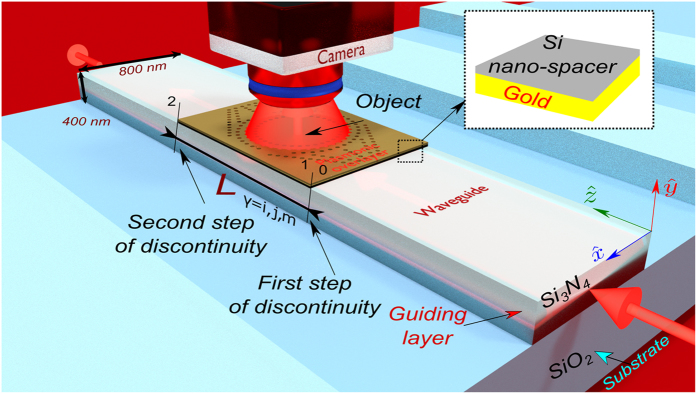
Illustration of the composite plasmonic waveguide structure and materials to study the invisibility cloaking scheme. Wavelength of *λ*_0_ = 637 nm illuminates the dielectric waveguide exciting the fundamental mode guided in region 0. Region 1 is characterized by the metasurface and Si nano-spacer placed on the waveguide with length *L* in the propagation direction exciting three hybrid plasmonic modes. Region 2 is identical to the region 0 in terms of the optical properties and functionality. A scattering object with optical index of 1.3 is placed on the metasurface.

Consider a guided wave system as shown in Fig. 1. A plasmonic metasurface with length of *L* = 10 μm, height of *d*_*m*_ = 40 nm is introduced on top of the silicon nitride^22^ (Si_3_N_4_) ridge waveguide to form a high index composite plasmonic waveguide structure on a silicon dioxide^23^ (SiO_2_) substrate. In order to confine the optical mode in vicinity with the metal-dielectric interface and for the efficient coupling to the surface plasmon modes, silicon^24^ (Si) nano-spacer is placed on top of the metasurface. The height of the Si nano-spacer is spatially varied in order to achieve control over the plasmonic wave. The composite plasmonic waveguide structure is confined in between the abrupt steps 1 and 2 and illuminated by the fundamental mode guided in the dielectric waveguide as shown in Fig. 1.

At the interface separating a dielectric with a permittivity *ε*_*d*_ and a metal with a permittivity *ε*_*m*_, SPs are evanescently excited by the coupling between free carriers of the metal and the incident electromagnetic field under momentum matching conditions. In composite plasmonic waveguide structure, the guided fundamental mode couples to the multiple modes excited in region 1 starting from the discontinuity with metasurface overlayer.

The use of CMOS technology compatible ridge waveguide^25^ in a combination with the metasurface^26–29^ provides design flexibility in on-chip light manipulations with novel SP based photonic circuits. The dielectric nano-spacer adds the additional degree of freedom for light confinement for coupling into hybrid plasmonic modes.

### Design of a Metamaterial Overlayer Using Transformation Optics

Although the general concepts of metamaterials were designed considering propogating waves, metasurfaces can be used to control waves in the near field regime, such as surface plasmons^30–34^. Here, we consider a transformation which maps rectangular region in the virtual system to an arbitrary region (the cloak) in the physical system.

As previously stated, the layer can potentially be electromagnetically transformed by changing the material properties of the medium. That means it is possible to change the coordinate system of the object, in order to obtain a new refractive index distribution which will present the same electromagnetic properties. Mathematically, this transformation can be realized by modifying the dielectric and magnetic properties of the materials described by the following relations:

1 $${\varepsilon }^{^{\prime} }={\bf{A}}\varepsilon {{\bf{A}}}^{T}/det({\bf{A}}),$$

2 $${\mu }^{^{\prime} }={\bf{A}}\mu {{\bf{A}}}^{T}/det({\bf{A}}),$$

where *ε* and *μ* are the dielectric and magnetic constants in the original space, and *ε*′ and *μ*′ are the same constants in the transformed one. **A** is the Jacobian transformation matrix which relates the coordinates between the physical and virtual systems and given by

3 $${\bf{A}}=[\begin{array}{c}\begin{array}{ccc}{\rm{\partial }}{x}^{^{\prime} }/{\rm{\partial }}x & {\rm{\partial }}{x}^{^{\prime} }/{\rm{\partial }}y & {\rm{\partial }}{x}^{^{\prime} }/{\rm{\partial }}z\\ {\rm{\partial }}{y}^{^{\prime} }/{\rm{\partial }}x & {\rm{\partial }}{y}^{^{\prime} }/{\rm{\partial }}y & {\rm{\partial }}{x}^{^{\prime} }/{\rm{\partial }}z\\ {\rm{\partial }}{z}^{^{\prime} }/{\rm{\partial }}x & {\rm{\partial }}{z}^{^{\prime} }/{\rm{\partial }}y & {\rm{\partial }}{z}^{^{\prime} }/{\rm{\partial }}z\end{array}\end{array}].$$

Where (*x*, *y*, *z*) are the coordinates of the physical space and (*x*′, *y*′, *z*′) are of the virtual space. To simplify the design, we assume that *μ*′ = *μ* = 1 symmetry along the *x* axis and consider a 2 dimensional transformation in the *x* − *z* plane with no change along the *y* axis. If the mapping satisfies the Cauchy–Riemann conditions^35^ which for our case are given by

3a $$\partial x^{\prime} /\partial x=\partial z^{\prime} /\partial z,$$

3b $$\partial x^{\prime} /\partial z=-\partial z^{\prime} /\partial x,$$

the transformed material becomes inhomogeneous and isotropic.

To generate the discrete coordinate transformation, the boundaries of the physical domain are first defined by

4 $$-5\le z\le 5\quad {\rm{a}}{\rm{n}}{\rm{d}}\quad \{\begin{array}{cc}0.2{\cos }^{2}{(\pi z/4)}^{2}\le x\le 0.4 & -2\le z\le 2\\ 0\le x\le 0.4 & {\rm{o}}{\rm{t}}{\rm{h}}{\rm{e}}{\rm{r}}{\rm{w}}{\rm{i}}{\rm{s}}{\rm{e}}\end{array}.$$

**Figure 2:**
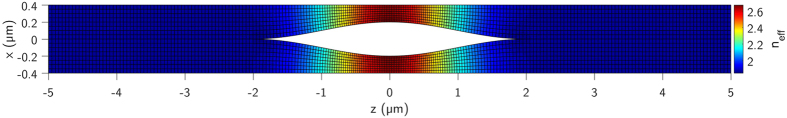
Transformed mesh using quasi-conformal transformation theme (black mesh) and calculated effective mode index, *n*_eff_.

To satisfy (3), the bounded area is divided into a mesh with 235 × 9 blocks and mapped using quasi-conformal (QC) transformation scheme^36^. The grid is numerically generated using an iterative algorithm that solves the discrete partial ecliptic differential equations generated by substituting (3) into (2) using successive over-relaxation (SOR) method^37^. The resulting orthogonal domain is then replicated in the bottom half space (−0.4 ≤ *x* ≤ 0) and represented by the black mesh as shown in Fig. 2.

Since the mesh in Fig. 2 is orthogonal and satisfies (3), the permittivity tensor in (1) becomes a scalar matrix and the effective mode index is ${n}_{{\rm{e}}{\rm{f}}{\rm{f}}}=\sqrt{{\varepsilon }^{^{\prime} }}=\sqrt{\varepsilon }/det({\bf{A}})$, represented by the color-map in Fig. 2.

The proposed method can conceal an object of an arbitrary shape if object’s longest dimension is less than the white cloaking area shown in Fig. 2. In addition, we found that our concept is feasible for the objects made of different materials such as dielectrics or metals.

## Results and Discussion

### Modal Distribution

**Figure 3:**
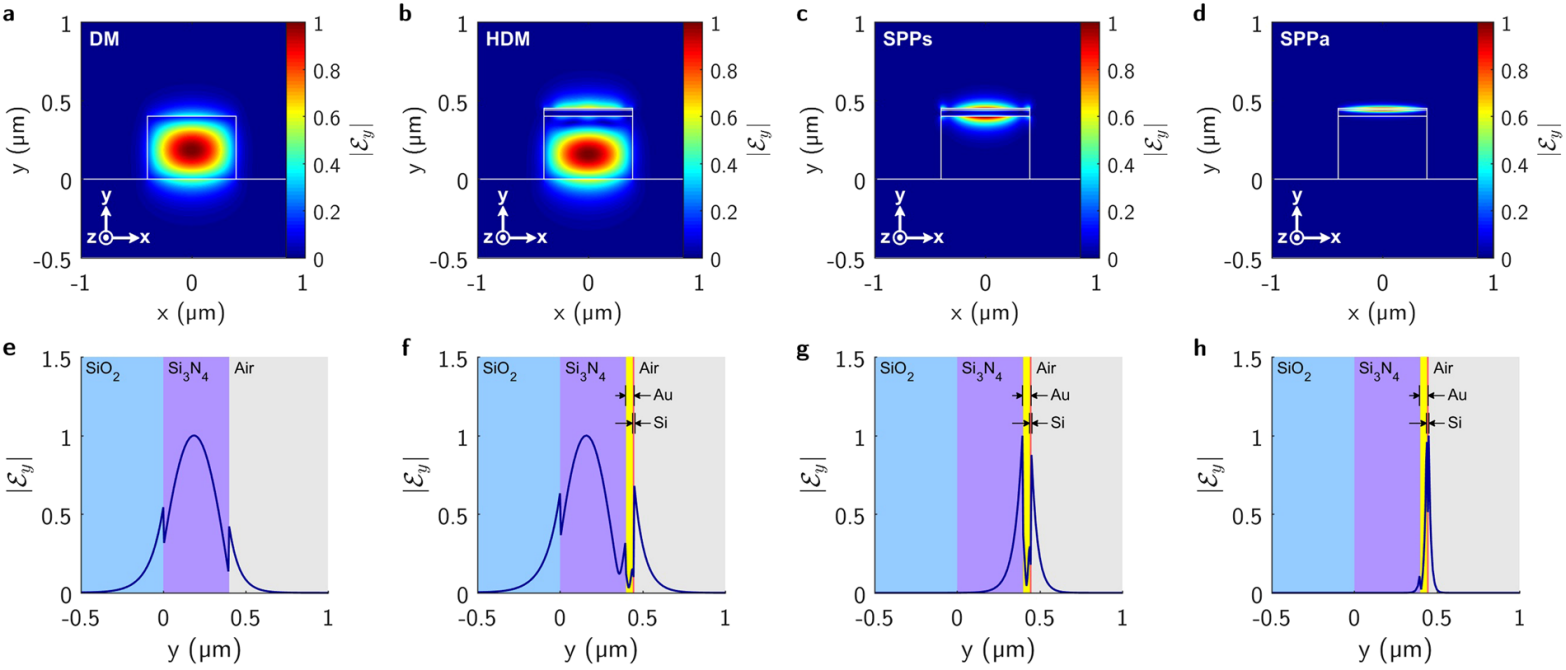
(Up) Calculated spatial distributions of *y*-component of the electric field magnitude $|{ {\mathcal E} }_{y}(x,y)|$ supported by the waveguide. (Down) Calculated electric field magnitude cross-sections $|{ {\mathcal E} }_{y}(x\,=\,\mathrm{0,}\,y)|$. (**a**) and (**e**) Show dielectric mode (DM), (**b**) and (**f**) show hybrid plasmonic/dielectric mode (HDM), (**c**) and (**g**) show SPP symmetric mode(SPP_s_); (**d**) and (**h**) show SPP asymmetric mode (SPP_a_).

Figure 3 shows the dominant *y*-component of the electric field magnitude supported by the waveguide for Si nano-spacer height of 10 nm. Figure 3(a) and Fig. 3(e) show the field profile of the purely dielectric mode (DM) supported by the dielectric waveguide in the $z < -L\mathrm{/2}$ and $z > L\mathrm{/2}$ regions. Figure 3(b) anf Fig. 3(f) show the profile of the fundamental hybrid mode supported by the composite dielectric/plasmonic waveguide for $-L\mathrm{/2}\, < \,z\, < \,L\mathrm{/2}$. HDM results as a combination of DM and a symmetric SPP mode supported by the adjacent thin metal ridge waveguide. Figure 3(c) and (g) show the profiles of the SPP_s_ and Fig. 3(d,h) show the SPP_a_ modes, supported by the thin metal ridge waveguide.

The nature of the modes depends on the optical properties of a waveguide structure and materials. They are localized in different areas of the waveguide. The region 1 is illuminated by the DM mode, which is the fundamental guided mode supported by the waveguide. Between the steps of discontinuity shown in Fig. 1, a resonance occurs when the DM matches the SPP_s_, together they create the HDM. SPP_a_ mode is a short range surface plasmon mode and compared to the symmetric mode, the asymmetric SPP_a_ mode penetrates much deeper to the metal film.

### Mechanism of the invisibility cloak with composite plasmonic waveguides

**Figure 4:**
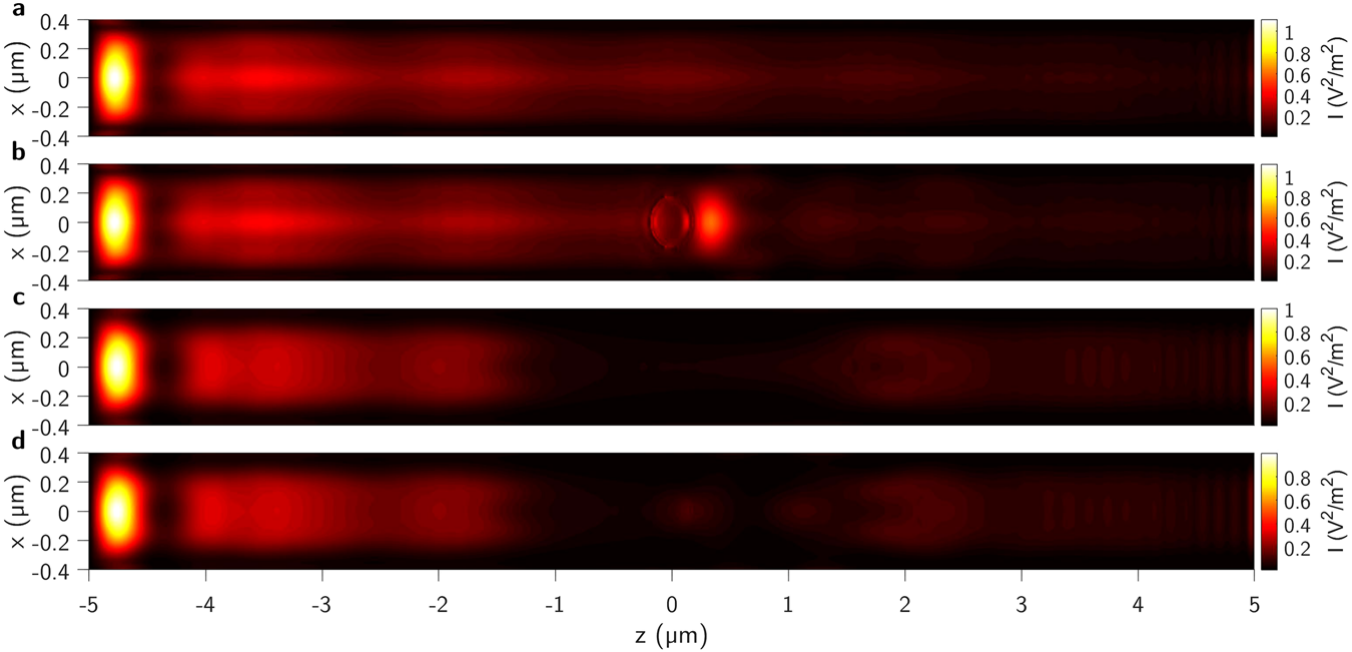
Calculated spatial surface intensities $|{ {\mathcal E} }_{y}(x,z{)|}^{2}$ at $y={y}_{s}$ in the composite plasmonic waveguide: (**a**) with slab gold overlayer, (**b**) with slab gold overlayer an object index of 1.3, (**c**) with transformed metasurface and (**d**) with transformed metasurface and an object.

As was described earlier, we study the cloak structure with composite plasmonic waveguides. The fundamental dielectric mode illuminates the waveguide overlapped with a metasurface of the effective permittivity. To confirm the cloak operation we calculate the integrated total surface intensity *I* over a waveguide width and along the interaction length *L* in the propagation direction (5) as shown on Fig. 4 while *I* is:

5 $$I={|\sum _{\eta =x,y,z}{E}_{\eta }(x,{y}_{s},z)|}^{2}={|\sum _{\eta =x,y,z}\sum _{\gamma =i,j,m}{c}_{i0,\gamma 1}{{\mathscr{E}}}_{\eta ,\gamma 1,d}(x,{y}_{s},z)|}^{2}.$$

${ {\mathcal E} }_{\gamma 1}(x,y,z)$ are extracted complex vectorial electric and magnetic field components calculated using FDTD. With the surface intensity *I* integrated across the plasmonic overlayer along the length from $z=-L\mathrm{/2}$ to $z=L\mathrm{/2}$ and full width of the waveguide. $i,j,m$ indicate guided modes which are HDM, SPP_s_ and SPP_a_ respectively as in^19^, ${c}_{i\mathrm{0,}\gamma 1}$ is the expansion coefficient and *η* is the electric field components in *x*, *y*, *z* directions.

Integrated surface intensity is an essential parameter to assess the effectiveness of evanescent invisibility cloak with a composite plasmonic waveguide. Figure 4 shows 3D colormaps of $|{E}_{\eta }{|}^{2}$ for four different cases. Integrated surface intensity of the composite plasmonic waveguide with slab gold overlayer is shown on Fig. 4a. Figure 4b shows *I* distribution calculated on composite plasmonic waveguide with slab gold overlayer while an object of cylindrical shape and index of 1.3 is placed on it. The object boundaries are visible due to the scattering effect as a result of the interaction with evanescent fields. Around the object, HDM shows strong localization across the metamaterial overlayer which results in observed increased intensity. Here, we are aiming to prevent the field localization around an object. Waveguide with transformed metamaterial overlayer is shown on Fig. 4c and with transformed metamaterial overlayer together with the object on Fig. 4d. Due to the carefully designed invisibility cloak on a composite plasmonic waveguide, the scattering effect from the object is avoided and invisibility is demonstrated.

The current bottleneck of realization of an invisibility cloak is the implementation of the required parameters, especially at visible wavelengths. Here we propose to spatially change the effective mode index of HDM mode by changing the thickness of the Si nano-spacer instead of spatially modifying the refractive index of the gold film. Correlating the properties of the structure with the calculated gradient index distribution can be physically realized by using grey-scale lithography technique^38,39^. This technique enables to adiabatically tailor the topology of the dielectric layer, in our case the Si nano-spacer height, adjacent to the gold surface to demonstrate the cloaking effect.

The fabrication procedure of the proposed design is straightforward. The first step is manufacturing Si_3_N_4_ ridge waveguides on SiO_2_ substrate by etching a slab of Si_3_N_4_ to the required dimensions. Then, a Poly(methyl methacrylate) (PMMA) photoresist is spin coated on the Si_3_N_4_ ridge and the pattern is created using electron beam (e-beam) lithography. The gold is deposited on the area exposed to the e-beam and then the Si thin film is deposited on top of the gold. For the creation of the specific pattern required for the device, the varying thickness of the Si nano-spacer is achieved using lift-off process.

**Figure 5:**
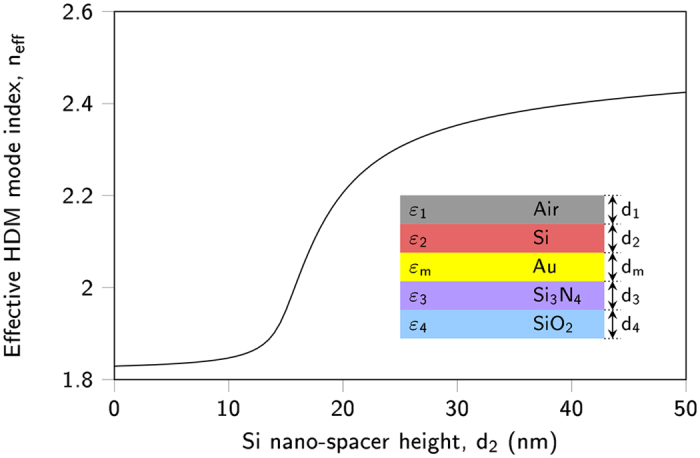
Calculated relation between Si nano-spacer height, *d*_2_, and effective mode index of HDM mode, *n*_eff_, at a wavelength of 637 nm. The structure consists of five layers: Air/Si/Au/Si_3_N_4_/SiO_2_, with their dielectric constants *ε*_1_/*ε*_2_/*ε*_*m*_/*ε*_3_/*ε*_4_ and widths *d*_1_/*d*_2_/*d*_*m*_/*d*_3_/*d*_4_, respectively.

Here, the proposed structure consists of five layers with their corresponding dielectric constants and widths, respectively, as shown in Fig. 5. The wave equation has to be satisfied in each distinct region, solving it results in an implicit expression for the dispersion relation linking *β* and *ω*. The effective mode index of HDM mode, defined as ${n}_{{\rm{eff}}}=\beta /{k}_{0}$, is obtained by numerically solving the structure for different heights of the Si layer as shown in Fig. 5.

The proposed device directs the flow of light smoothly around the cloaked region and is effective for a wide range of refractive indices and materials. We explored the effectiveness of the cloak in terms of the optical properties of the object. We found that the concealing effect is preserved for variety of materials which agrees with the concepts of transformation optics.

To conclude, here we proposed the new composite plasmonic waveguide scheme with dielectic nano-spacer based on the transformation optics principles to manipulate with light and distort the evanescent fields in a controllable manner to conceal an object. The plasmonic metasurface is placed on the composite plasmonic waveguide with the nano-spacer. High dielectric nano-spacer made of Si has contributed to the light confinement in vicinity with the metasurface boundary and facilitated the coupling to the hybrid plasmonic modes. The light manipulation is realized due to the engineered effective permittivity which in turn avoids the scattering effect. Our calculated results demonstrate that the designed metasurface can deflect the evanescent wave into a predefined, analytically calculated pattern. Since plasmons are localized in the direction perpendicular to the metamaterial overlayer boundary and accompanied by the combination of the transverse and longitudinal electromagnetic fields, they have maximum intensity on the surface with the metamaterial overlayer. The gradient material properties of this layer enable the invisibility effect to take place. Our theoretical study of invisibility cloaking scheme with composite plasmonic waveguides and Si nano-spacer enables the understanding of the complex behavior of hybrid plasmonic modes for the light manipulation on-chip and further design of integrated on-chip devices.

## Methods

### Expression of the eigenmodes at an abrupt step for the calculations of the surface intensity

One can achieve the modes at the beginning of the gold-coated region (1) and the fundamental mode excited at the input region (0) to determine the entire composite waveguide by applying appropriate mode-matching conditions at the input (*z* = 0) and output (*z* = *L*) interfaces of the dielectric rib waveguide and the composite dielectric/metallic stripe waveguide. We assume guided pure dielectric mode, DM, in Fig. 3(a) designated by a subscript $i0$ in the input: region 0 in Fig. 3 and output: Region 2 on Fig. 1 side (pure dielectric waveguide) of the first step and orthogonal guided modes $\gamma 1\,=\,i\mathrm{1,}\,j1$ or *m*1 assigned to HDM in cross-sections in Fig. 3(b), SPP_s_ in Fig. 3(c) and SPP_a_ in Fig. 3(d) modes, respectively, in the output side (dielectric waveguide with plasmonic overlayer) of the first step as shown in Fig. 1(a) with quasi-transverse magnetic components. At $z\,=\,0$, the general complex field distributions at the boundary between region 0 and region 1, ignoring the reflected and radiated modes, are:

6 $${E}_{\xi i0}=\sum _{\gamma =i,j,m}{E}_{\xi \gamma 1}\quad \,{\rm{and}}\quad {H}_{\xi i0}=\sum _{\gamma =i,j,m}{H}_{\xi \gamma 1}\mathrm{.}$$

Where $\xi =x,y$ and ${\bf{E}}={E}_{x}\hat{x}+{E}_{y}\hat{y}+{E}_{z}\hat{z}$ and ${\bf{H}}={H}_{x}\hat{x}+{H}_{y}\hat{y}+{H}_{z}\hat{z}$. $\hat{x}$, $\hat{y}$ and $\hat{z}$ are unit vectors in the *x*, *y* and *z* directions respectively. An expression for the expansion coefficient between input mode $i1$ in region 0 and mode $j1$ in region 1 is derived using the complex orthogonality principle:

7 $${\int }_{-{\rm{\infty }}}^{{\rm{\infty }}}{\int }_{-{\rm{\infty }}}^{{\rm{\infty }}}{({{\bf{E}}}_{i0}\times {{\bf{H}}}_{\gamma 1})}_{z}+{({{\bf{E}}}_{\gamma 1}\times {{\bf{H}}}_{i0})}_{z}{\rm{d}}{\rm{x}}{\rm{d}}{\rm{y}}={\int }_{-{\rm{\infty }}}^{{\rm{\infty }}}{\int }_{-{\rm{\infty }}}^{{\rm{\infty }}}{({{\bf{E}}}_{\gamma 1}\times {{\bf{H}}}_{\gamma 1})}_{z}+{({{\bf{E}}}_{\gamma 1}\times {{\bf{H}}}_{\gamma 1})}_{z}{\rm{d}}{\rm{x}}{\rm{d}}{\rm{y}}.$$

Where $\gamma 1=i\mathrm{1,}\,j\mathrm{1,}\,m1$. We now express a general complex electric and magnetic field distribution components:

8 $${{\bf{E}}}_{\delta }(x,y,z)={a}_{\delta }{\bar{{\mathscr{E}}}}_{\delta }(x,y)\exp (-j{\beta }_{\delta }z)$$

and

9 $${{\bf{H}}}_{\delta }(x,y,z)={a}_{\delta }{\bar{ {\mathcal H} }}_{\delta }(x,y)\exp (-j{\beta }_{\delta }z\mathrm{).}$$

Where ${\beta }_{\delta }$ is the propagation constant of mode $\delta$. ${\bar{ {\mathcal E} }}_{\delta }(x,y,z)$ and ${\bar{ {\mathcal H} }}_{\delta }(x,y,z)$ are extracted complex vectorial electric and magnetic field components calculated using FEM and $\bar{ {\mathcal E} }={ {\mathcal E} }_{x}\hat{x}+{ {\mathcal E} }_{y}\hat{y}+{ {\mathcal E} }_{z}\hat{z}$ and $\bar{ {\mathcal H} }={ {\mathcal H} }_{x}\hat{x}+{ {\mathcal H} }_{y}\hat{y}+{ {\mathcal H} }_{z}\hat{z}$. ${a}_{\delta }={N}_{\delta }{A}_{\delta }={E}_{\delta }(x,y,z)/({ {\mathcal E} }_{\delta }(x,y)exp(-j{\beta }_{\delta }z))={H}_{\delta }(x,y,z)/({ {\mathcal E} }_{\delta }(x,y)exp(-j{\beta }_{\delta }z))$ and ${A}_{\delta }$ is complex, ${A}_{\delta }=|{A}_{\delta }|exp(-j{\phi }_{\delta })$ related to the power carried by the mode as: ${P}_{\delta }=|{A}_{\delta }{|}^{2}$. The normalization factor ${N}_{\delta }$ giving rise to each mode carrying unity power, ${P}_{\delta }\,=\,1$ is: ${N}_{\delta }\,=\,{\mathrm{(2/}\Re ({\int }_{-\infty }^{\infty }{\int }_{-\infty }^{\infty }{({\bar{ {\mathcal E} }}_{\delta }\times {{\bar{ {\mathcal H} }}^{\ast }}_{\delta })}_{z}{\rm{dxdy}}))}^{\mathrm{1/2}}$. For *z* = 0 these are:

10 $${a}_{i0}{ {\mathcal H} }_{\xi i0}=\sum _{\gamma =i,j,m}{a}_{\gamma 1}{ {\mathcal H} }_{\xi \gamma 1};\,\,{a}_{i0}{ {\mathcal E} }_{\xi i0}=\sum _{\gamma =i,j,m}{a}_{\gamma 1}{ {\mathcal E} }_{\xi \gamma 1}\mathrm{.}$$

Power in any region is defined as:

11 $$P=\frac{1}{2}{\rm{\Re }}\{{\int }_{-{\rm{\infty }}}^{{\rm{\infty }}}{\int }_{-{\rm{\infty }}}^{{\rm{\infty }}}{(\bar{{\mathscr{E}}}\times {\bar{{\mathscr{H}}}}^{\ast })}_{z}{\rm{d}}{\rm{x}}{\rm{d}}{\rm{y}}\}.$$

A relation between eigenmodes at an abrupt step is detailed below.

By substituting (8) and (9) into (7) we obtain:

12 $${a}_{i0}{a}_{\gamma 1}{\int }_{-{\rm{\infty }}}^{{\rm{\infty }}}{\int }_{-{\rm{\infty }}}^{{\rm{\infty }}}{({\bar{{\mathscr{E}}}}_{i0}\times {\bar{{\mathscr{H}}}}_{\gamma 1})}_{z}+{({\bar{{\mathscr{E}}}}_{\gamma 1}\times {\bar{{\mathscr{H}}}}_{i0})}_{z}{\rm{d}}{\rm{x}}{\rm{d}}{\rm{y}}={a}_{\gamma 1}^{2}{\int }_{-{\rm{\infty }}}^{{\rm{\infty }}}{\int }_{-{\rm{\infty }}}^{{\rm{\infty }}}{({\bar{{\mathscr{E}}}}_{\gamma 1}\times {\bar{{\mathscr{H}}}}_{\gamma 1})}_{z}+{({\bar{{\mathscr{E}}}}_{\gamma 1}\times {\bar{{\mathscr{H}}}}_{\gamma 1})}_{z}{\rm{d}}{\rm{x}}{\rm{d}}{\rm{y}}.$$

To obtain a relation between eigenmodes at an abrupt step:

13 $${a}_{i0}({I}_{i0,\gamma 1}+{I}_{\gamma 1,i0})={a}_{\gamma 1}2{I}_{\gamma 1,\gamma 1},$$

and

14 $${a}_{\gamma 1}={a}_{i0}({I}_{i0,\gamma 1}+{I}_{\gamma 1,i0})/(2{I}_{\gamma 1,\gamma 1}).$$

Where:

15 $${I}_{i,\gamma }={\int }_{-{\rm{\infty }}}^{{\rm{\infty }}}{\int }_{-{\rm{\infty }}}^{{\rm{\infty }}}{({\bar{{\mathscr{E}}}}_{i}\times {\bar{{\mathscr{H}}}}_{\gamma })}_{z}{\rm{d}}{\rm{x}}{\rm{d}}{\rm{y}}={\int }_{-{\rm{\infty }}}^{{\rm{\infty }}}{\int }_{-{\rm{\infty }}}^{{\rm{\infty }}}{({{\mathscr{E}}}_{xi}{{\mathscr{H}}}_{y\gamma }-{{\mathscr{E}}}_{yi}{{\mathscr{H}}}_{x\gamma })}_{z}{\rm{d}}{\rm{x}}{\rm{d}}{\rm{y}}.$$

${N}_{\delta }$ can then be expressed as:

16 $${N}_{\delta }={(2/{\rm{\Re }}({I}_{\delta ,\delta }))}^{1/2},$$

which is:

17 $${A}_{\gamma 1}{N}_{\gamma 1}={A}_{i0}{N}_{i0}({I}_{i0,\gamma 1}+{I}_{\gamma 1,i0})/(2{I}_{\gamma 1,\gamma 1}).$$

An expansion coefficient ${c}_{i\mathrm{0,}\gamma 1}$ expanding mode *i*1 from region 0 into mode *γ*1 in region 1 over the first abrupt step is:

18 $${c}_{i0,\gamma 1}={a}_{\gamma 1}/{a}_{i0}={N}_{i0}({I}_{i0,\gamma 1}+{I}_{\gamma 1,i0})/(2{I}_{\gamma 1,\gamma 1}{N}_{\gamma 1}).$$

At *z* = *L*, the expansion coefficients are derived in a similar manner to that detailed above resulting in:

19 $${c}_{\gamma 1,i2}=({I}_{\gamma 1,i2}+{I}_{i2,\gamma 1})\,\exp \,[-j({\beta }_{\gamma 1}-{\beta }_{i2})L]/(2{I}_{i2,i2}).$$

Since,

$${a}_{\gamma 1}={c}_{i\mathrm{0,}\gamma 1}{a}_{i0},$$

20 $${a}_{i2}={c}_{i\mathrm{0,}\gamma 1}{a}_{i0}{c}_{\gamma \mathrm{1,}i2}$$

or:

21 $${A}_{i2}{N}_{i2}={c}_{i\mathrm{0,}\gamma 1}{A}_{i0}{N}_{i0}{c}_{\gamma \mathrm{1,}i2}\mathrm{.}$$

And the transmittance through the composite plasmonic waveguide is obtained as:

22 $$T(z=L)={|{A}_{i2}/{A}_{i0}|}^{2}={|\sum _{\gamma =i,j,m}{c}_{i0,\gamma 1}{c}_{\gamma 1,i2}({N}_{i0}/{N}_{i2})|}^{2},$$

or:

23 $$T={|{\sum }_{\gamma 1=i,j,m}{C}_{\gamma 1}\exp (-i{\alpha }_{\gamma 1}L)|}^{2}.$$

Where ${C}_{\gamma 1}=({I}_{i\mathrm{0,}\gamma 1}+{I}_{\gamma \mathrm{1,}i0}{)}^{2}\mathrm{/(4}{I}_{i\mathrm{0,}i0}{I}_{\gamma \mathrm{1,}\gamma 1})$ and *L* is length of a gold overlayer.

The transmittance through the composite-plasmonic waveguide structure presented here, can be calculated using (22) (or (23)).

### Simulation of invisibility cloak on a composite plasmonic waveguide

The effective permittivity was derived and implemented using MATLAB. The simulations were performed using the commercial software packages Lumerical FDTD. In calculations, the fundamental mode was used as a source to excite hybrid plasmonic modes. The guided modes were explored using mode monitor. The distribution of the surface intensity was explored using field monitor while recorded simultaneously. Perfectly matched layers (PMLs) were used as the boundary conditions at the *z* axis and symmetry boundary conditions were used along the *y* axis. The simulation mesh was set to the high accuracy with the mesh step of 0.01 *μ*m. The mesh around the gold and silicon layers was set to finer mesh with steps of 0.0025 μm. Conformal mesh refinement was applied to all materials. Lumerical FDTD simulations and MATLAB calculations were performed on a workstation with the following components: Intel Xenon 3.5 GHz E5-1650 CPU with 32 GB of RAM.
